# Benefits of clinical criteria and high-throughput sequencing for diagnosing children with syndromic craniosynostosis

**DOI:** 10.1038/s41431-020-00788-4

**Published:** 2020-12-07

**Authors:** Elin Tønne, Bernt Johan Due-Tønnessen, Inger-Lise Mero, Ulrikke Straume Wiig, Mari Ann Kulseth, Magnus Dehli Vigeland, Ying Sheng, Charlotte von der Lippe, Kristian Tveten, Torstein Ragnar Meling, Eirik Helseth, Ketil Riddervold Heimdal

**Affiliations:** 1 Faculty of Medicine, University of Oslo, Oslo, Norway; 2 Department of Medical Genetics, Oslo University Hospital, Oslo, Norway; 3 Norwegian National Unit for Craniofacial Surgery, Oslo University Hospital, Oslo, Norway; 4 Department of Neurosurgery, Oslo University Hospital, Oslo, Norway; 5 Centre for Rare Disorders, Oslo University Hospital, Rikshospitalet, Oslo, Norway; 6 Department of Medical Genetics, Telemark Hospital Trust, Skien, Norway; 7 Faculty of Medicine, University of Geneva, Geneva, Switzerland; 8 Department of Neurosurgery, Geneva University Hospitals, Geneva, Switzerland

**Keywords:** Genetic testing, Genetics research, Diseases

## Abstract

An accurate diagnosis of syndromic craniosynostosis (CS) is important for personalized treatment, surveillance, and genetic counselling. We describe detailed clinical criteria for syndromic CS and the distribution of genetic diagnoses within the cohort. The prospective registry of the Norwegian National Unit for Craniofacial Surgery was used to retrieve individuals with syndromic CS born between 1 January 2002 and 30 June 2019. All individuals were assessed by a clinical geneticist and classified using defined clinical criteria. A stepwise approach consisting of single-gene analysis, comparative genomic hybridization (aCGH), and exome-based high-throughput sequencing, first filtering for 72 genes associated with syndromic CS, followed by an extended trio-based panel of 1570 genes were offered to all syndromic CS cases. A total of 381 individuals were registered with CS, of whom 104 (27%) were clinically classified as syndromic CS. Using the single-gene analysis, aCGH, and custom-designed panel, a genetic diagnosis was confirmed in 73% of the individuals (*n* = 94). The diagnostic yield increased to 84% after adding the results from the extended trio-based panel. Common causes of syndromic CS were found in 53 individuals (56%), whereas 26 (28%) had other genetic syndromes, including 17 individuals with syndromes not commonly associated with CS. Only 15 individuals (16%) had negative genetic analyses. Using the defined combination of clinical criteria, we detected among the highest numbers of syndromic CS cases reported, confirmed by a high genetic diagnostic yield of 84%. The observed genetic heterogeneity encourages a broad genetic approach in diagnosing syndromic CS.

## Introduction

Craniosynostosis (CS) is one of the most common inborn anomalies in children, affecting 1/1600–1/1800 live births [1, 2]. CS is classified into syndromic and nonsyndromic CS, where syndromic CS is reported to constitute 12–31% of all cases [3–5]. Individuals with syndromic CS have an increased risk of additional complications and repeat craniofacial surgery [6, 7], and need to be identified. Hence, an accurate molecular diagnosis is important for personalized treatment and surveillance, in addition to genetic counselling, family planning, social care, and support from patient organizations.

Previously, syndromic CS was defined by the occurrence of one of the frequent and well-known syndromes: Apert, Muenke, Saethre–Chotzen, Pfeiffer, or Crouzon, caused by genetic variants in the *FGFR2*, *FGFR3*, *TWIST1*, *FGFR1/2*, and *FGFR2* genes, respectively [1, 8]. High-throughput sequencing (HTS) has improved and changed the diagnostics of syndromic CS over the last two decades, and genetic variants in at least 80 genes are known to cause syndromic CS [9, 10].

There is no clear consensus regarding the definition of syndromic CS. Some studies limit their cohort to a defined selection of verified genetic diagnoses [11, 12], whereas others limit their cohort to affected sutures only, as complex or coronal synostoses are more commonly associated with syndromic CS [12, 13], or use a combination of clinical criteria [5, 14]. A recent population-based epidemiological study from our group demonstrated a high proportion of syndromic cases of 27% defined by clinical criteria and a genetic detection rate of 75% after testing with array comparative genomic hybridization (aCGH) and exome-based HTS, filtering for 72 genes associated with syndromic CS [2]. We detected many midline synostoses in individuals with syndromic CS, in particular in individuals with rare genetic syndromes [2], suggesting that an affected suture alone does not provide sufficient evidence to determine whether an individual has syndromic or nonsyndromic CS. We hypothesized that a broader approach to genetic testing would further increase the diagnostic yield.

In this study, all individuals with syndromic CS born between 1 January 2002 and 30 June 2019, selected by clinical criteria, and registered in the registry of the Norwegian National Unit for Craniofacial Surgery were included. Supplemental genetic diagnostics of HTS filtering for a panel of 1570 genes informed by the Deciphering Developmental Delay study (DDG2P) were offered for negative cases. We present a large variety of genetic syndromes and aim to propose a strategy for clinical classification and genetic testing of individuals with syndromic CS.

## Materials and methods

**Fig. 1:**
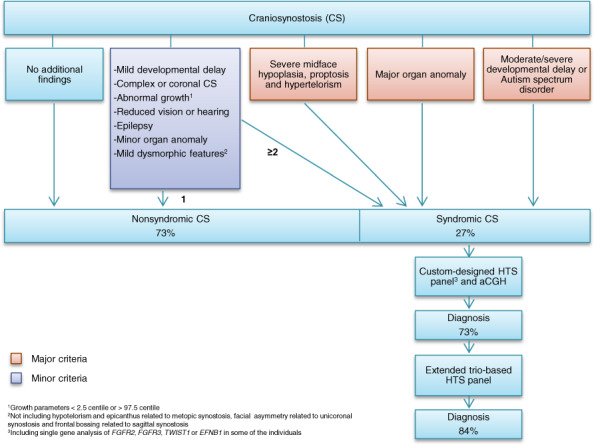
Flow chart showing clinical criteria and genetic analysis of syndromic CS. Minor criteria are presented in the dark blue panel and major criteria in the red panels. Syndromic CS is defined by the addition of two or more minor criteria or one major criterion.

The study was approved by the Norwegian Regional Committees for Medical and Health Research Ethics (REK_2018/797) and by Oslo University Hospital (permit number P360:18/05374). Informed consent was obtained from all individuals that participated in the study. Since 2001, all individuals in Norway with suspected CS have been referred to the Norwegian National Unit for Craniofacial Surgery at Oslo University Hospital for diagnostics, treatment, and follow-up [2]. Individuals suspected of having syndromic CS are seen regularly by the unit’s multidisciplinary team, including a clinical geneticist. The unit’s registry is prospective and includes all consenting individuals diagnosed with CS (85%) [2]. Individuals with CS born between 1 January 2002 and 30 June 2019 and registered by 23 October 2019 were included in the study (*n* = 381). The database was updated January 2020 to include the latest genetic results. Syndromic CS was defined by a combination of clinical criteria, formulated by the authors, with one major criterion or two or more minor criteria; details are presented in Fig. 1. All individuals were classified by the same two clinical geneticists prior to inclusion (ET and KRH).The genetic analyses were offered stepwise. Individuals suspected of having one of the common and well-described CS syndromes were initially tested by single-gene analysis of *FGFR2*, *FGFR3*, *TWIST1* or *EFNB1*. If the results came back negative, aCGH was performed. When the clinical presentation did not resemble one of the common CS syndromes, aCGH was offered initially. From 2016, exome-based HTS filtering for a custom-designed panel of 72 genes associated with syndromic CS (Supplemental Table 1) was performed if the result of the aCGH came back negative. If this did not result in a genetic diagnosis the extended trio-based HTS panel of 1570 genes was offered. A few individuals (*n* = 6) were diagnosed prior to assessment by the unit’s team. Their findings are presented in the results section under the diagnostic tool in which they would have been found in the stepwise approach (Tables 2–4). Ten individuals were excluded from the calculations of diagnostic yield, because they did not want genetic testing (*n* = 4), and were analysed with aCGH only (*n* = 5) or with single gene and aCGH only (*n* = 1). Individuals analysed with aCGH and HTS filtering for the custom-designed panel only (*n* = 3) were included in the calculations. All individuals with nonsyndromic CS of the coronal suture(s), or with an affected first-degree relative, were offered the custom-designed HTS panel due to the risk of having a monogenetic cause (e.g., *TCF12*). As genetic causes of nonsyndromic CS is not the scope of this study, these results are not included. Blood samples were obtained from all patients, followed by DNA extraction with QiaSymphony DSP DNA Mini Kit (Qiagen, Cologne, Germany). For Sanger sequencing of *FGFR2*, *FGFR3*, *TWIST1*, and *EFNB1*, primers were designed using primer3 software, sequencing was done on an ABI 3730 sequencer (Applied Biosystems, Life Technologies, CA, USA), and sequence data were analysed using SeqScape v2.7 (Life Technologies, CA, USA). For MLPA of *TWIST1*, the Salsa MLPA Probemix P054 (MRC Holland) was used. Array CGH was performed using Agilent 180 K SurePrint G3 Human CGH (Agilent Technologies, Santa Clara, CA, USA) according to the manufacturer’s recommendations. Data were processed with Feature Extraction and DNA Analytics (Agilent Technologies). Exome-based HTS was performed by using Agilent SureSelect^XT^ Target Enrichment 50 Mb Kit (Agilent Technologies, Santa Clara, CA, USA) for library preparation and Illumina HiSeq 2500 in high‐output run mode. Bioinformatic handling of the sequencing data followed the practice from Genome Analysis Tool Kit for exome sequencing [15]. Raw reads were mapped to the reference sequence (GRCh37/hg19). Joint variant calling was performed within each trio. Variant annotation was done by Annovar [16]. Downstream filtering and analysis were done with Filtus [17] on the variants within coding regions and intron/exon boundaries of the custom-designed panel or the extended trio-based panel of 1570 genes. The extended trio-based panel was informed by the Deciphering Developmental Disorders study (DDG2P) [18] and was the largest panel available at our laboratory. We selected variants with allele frequency of less than 0.5% (for genes inherited as autosomal dominant) or less than 1% (for other inheritance patterns), as reported in gnomAD [19]. Variants were classified according to the guidelines by the American College of Medical Genetics and Genomics [20], and only class 4 (likely pathogenic) and class 5 (pathogenic) variants were included in the results. All variants were submitted to ClinVar (SCV001437545–SCV001437592).

## Results

**Fig. 2:**
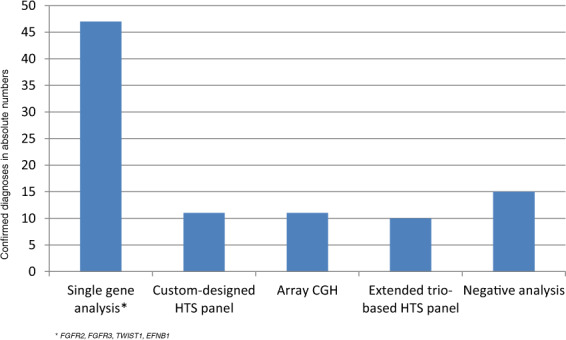
Confirmed genetic diagnoses by method. The distribution of confirmed diagnoses is given in absolute numbers.

In total, 381 individuals were registered with CS, of which 104 (27%) were clinically classified as syndromic based on the criteria presented in Fig. 1. A total of 94 individuals with syndromic CS (90%) accepted the stepwise genetic testing presented in the method section. By single-gene analysis, aCGH and the custom-designed panel, a genetic diagnosis was confirmed in 69 individuals (73%; Figs. 1 and 2). When including the results of the extended trio-based HTS panel, the number of genetically confirmed diagnoses increased to 79 (84%; Figs. 1 and 2, Supplemental Table 2). When excluding the CS syndromes caused by variants in the *FGFR2*, *FGFR3*, *TWIST1*, or *EFNB1* gene, a genetic cause was confirmed in 26 individuals (28%), partitioned into 23 different genetic or chromosomal causes, 16 of these not commonly associated with CS (Tables 1–4). Fifteen individuals (16%) had negative genetic test results (Fig. 2).

**Table 1:** Genetically confirmed diagnoses by single-gene analysis (Sanger sequencing).

Syndrome	Gene	Cases	Male/female	Suture^a^	Familial^b^
Apert	*FGFR2*	15	6/9	BC, LCS, MS	0
Muenke	*FGFR3*	14	7/7	BC, RC	8 (6 index)
Saethre–Chotzen	*TWIST1*	8	4/4	BC, LC, RC,	6 (4 index)
Crouzon/Pfeiffer/Beare–Stevenson syndrome	*FGFR2*	5	2/3	BC, BL, BCBL, P, S	1
Crouzon with acanthosis nigricans	*FGFR3*	3	1/2	BCS, P	0
Craniofrontonasal dysplasia	*EFNB1*	2	0/2	BC, RC	0

*BC* bicoronal, *BCBL* bicoronal and bilambdoid, *BCS* bicoronal and sagittal, *BL* bilambdoid, *LC* left coronal, *LCS* left coronal and sagittal, *MS* metopic and sagittal, *P* pancynostosis, *RC* right coronal, *S* sagittal.

^a^Affected suture: BC, BCBL, BL, BCS, LC, LCS, MS, P, RC, S.

^b^Individuals with an affected first- or second-degree relative.

Fifty-three individuals (56%) had variants in one of the genes frequently associated with CS syndromes (*FGFR2*, *FGFR3, TWIST1*, and *EFNB1*). Of these, 47 individuals (89%) had a clinical phenotype in concordance with the genetic diagnosis and were diagnosed by single-gene analysis (Table 1).

Ten individuals (11%) had a de novo copy number variation associated with a known microdeletion or duplication syndrome (Fig. 2); seven of these are not commonly associated with CS (Table 2). In addition, one case of Saethre–Chotzen syndrome, caused by a deletion including the *TWIST1* gene, was detected by aCGH (Table 2).

**Table 2:** Chromosome aberrations in individuals with syndromic CS.

	Chromosome aberration^a^	Position^b^	Size (MB)	Candidate gene	Diagnosis	Male/female	Suture^c^	Clinical phenotype
Chromosome aberrations not commonly associated with CS	Del 17p13.3	g.84287_2468384del	2.4	*CRK* *YWHAE*	17p13.3 Microdeletion syndrome (without *PAFAH1B1* deletion)	M	P	Development delay, short stature, hypotonia, reduced vision
	Del 1p32.3p31.3	g.53675707_ 66644963del	13	*NFIA*	1p32-p31 Deletion syndrome	M	M	Intrauterine growth restriction, developmental delay, preaxial polydactyly, inguinal hernia, short stature, corpus callosum agenesis, optic nerve hypoplasia, thoracic hypoplasia, hearing loss, microphthalmia, micrognathia, dysplastic ears
	Dup 22q11.1q12.1	g.16888899_26483608dup	9.6	No candidate gene	Cat Eye syndrome and 22q11.1q12.1 microduplication syndrome	M	M	Developmental delay, ASD, reduced vision, torticollis, micrognathia, hypotelorism, epicanthus
	Del 1p22.1	g.92405898_ 94018197del	1.6	*RPL5*	Diamond–Blackfan anaemia, 6	M	S	Developmental delay, AVSD, severe feeding difficulties, anaemia, short stature, long philtrum, thin upper lip, proximal thumb
	Del 2q37.1q37.3 Dup 11p15.5p15.4 mat	g.233110452_ 243028452del g.210300_ 8664358dup	10 8.5	*HDAC4*	2q37 Deletion syndrome and Silver–Russell syndrome	F	RC	Developmental delay, respiratory distress, cardiomegaly, hypotonia, midface hypoplasia, epicanthus, died at 12 months of age
	Del 2q24.2q31.3^d^	g.163078055_ 182119617del	19	No candidate gene	2q24 Deletion syndrome	M	BL, LC, S	Developmental delay, VSD, epilepsy, finger contractures, syndactyly, proptosis, hypertelorism, died at 12 months of age
	Del 6q16.2q21	g.98949950_114533905del	16	No candidate gene	6q15-6q23 deletion syndrome	F	M	Developmental delay, reduced vision, respiratory distress
Chromosome aberrations commonly associated with CS	Del 7p15.3p21.2	g.14470668_20385165del	6	*TWIST1*	Saethre–Chotzen syndrome	F	LC	Normal development, facial asymmetry, low frontal hairline, small rounded ears, brachydactyly, scoliosis, father mosaic
	Del 9pterp22.2	g.204193_ 18073357del	17.8	*FREM1*	9p Deletion syndrome	F	M	Developmental delay, epilepsy, omphalocele, reduced vision
	Del 9p23p22.1	g.13638428_ 17121764del	3.5	*FREM1*	9p Deletion syndrome	M	M	Developmental delay, reduced vision
	Dup 5q35.1q35.3^d^	g.170805664_ 180719789dup	10	*MSX2* *NSD1*	5q35 Duplication syndrome	M	S	Developmental delay, VSD, midface hypoplasia, hypotelorism

*BC* bicoronal, *BL* bilambdoid, *LC* left coronal, *M* metopic, *P* pancynostosis, *RC* right coronal, *S* sagittal.

^a^NCBI_Build 37 (hg19).

^b^Inner start-stop coordinate.

^c^Affected suture: BC, BL, LC, M, P, RC, S.

^d^Analysis performed at an external laboratory.

Of the 11 individuals diagnosed by the custom-designed HTS panel, seven had clinical phenotype in concordance with their genetic diagnosis, while four had unexpected clinical presentation (Table 3). A girl with an *EFNB1* variant had a complex heart malformation not associated with craniofrontonasal syndrome. A boy with classic features of craniofrontonasal syndrome was not analysed by single-gene analysis due to his gender. However, HTS revealed that he was mosaic for a variant in the *EFNB1* gene (Table 3) and karyotyping confirmed XY, male. In a boy with Crouzon-like appearance, with negative result of a *FGFR2* analysis, HTS detected a variant in *TWIST1* consistent with Saethre–Chotzen syndrome. A homozygous variant in *IL11RA* consistent with CS and dental anomalies syndrome was detected in a girl with late-occurring pansynostosis (4 years old) and no dental anomalies (Table 3). In addition, we detected two cases of parental mosaicism for variants in *FGFR2* and *ZIC1*, respectively, both associated with autosomal dominant inheritance (Table 3). The individuals with the *FGFR2* variant were siblings and not analysed by single-gene analysis due to the suspicion of autosomal recessive inheritance.

**Table 3:** Genetically confirmed diagnoses by the custom-designed HTS panel, divided by expected and unexpected clinical presentation.

	Syndrome	Gene	Variant	Inheritance	Male/female	Suture^b^	Clinical features
Expected clinical presentation	Craniosynostosis and dental anomalies	*IL11RA* NM_001142784.2	c.781 C > T p.(Arg261Cys)	Recessive	M	P	Chiari I malformation, microcephaly, midface hypoplasia, Crouzon-like appearance
	Cranioectodermal dysplasia/Sensenbrenner	*IFT122* NM_052985.1	c.1118 C > T p.(Ser373Phe)	Recessive	M	S	Renal failure, sensorineural hearing deficit, short statue, telecanthus, micrognathia tooth anomalies, brachydactyly, Tourette syndrome
	Craniosynostosis 4 CRS4	*ERF* NM_006494.2	c.1201_1202del p.(Lys401Glufs*10)	Dominant, de novo	F	S	Midface hypoplasia, hypertelorism, short nose
	Craniosynostosis 3 CRS3	*TCF12* NM_207036.1	c.778_779del p.(Met260Valfs*5)	Dominant, paternal	M	LC	Low anterior hairline, brachydactyly, transverse palmar crease, healthy father
	Craniosynostosis 6 CRS6	*ZIC1*^a^ NM_003412.3	c.1153 G > T p.(Glu385*)	Dominant, maternal mosaic	M	BC, LL	Developmental delay, severe speech delay, reduced vision, proptosis, midface hypoplasia, tubular nose, healthy mother
	Crouzon	*FGFR2* NM_000141.4	c.824_829dup p.(Glu275_Phe276dup)	Dominant, paternal mosaic	M/F	BC,S	Two siblings with typical Crouzon phenotype, healthy father
Unexpected clinical presentation	Craniofrontonasal dysplasia	*EFNB1* NM_004429.4	c.182 A > G p.(Asp61Gly)	De novo, mosaic	M	RC	Short and asymmetric skull, hypertelorism, broad and depressed nasal root, asymmetric eyes, widow’s peak, pectus excavatum, dysplastic nails
	Craniofrontonasal dysplasia	*EFNB1* NG_008887.1 (NM_004429.4)	c.128 + 5 G > A splice	Dominant, de novo	F	LC	Atrial septal defect (ASD), facial asymmetry, hypertelorism, broad nasal root, bifid nasal tip, widow’s peak
	Craniosynostosis and dental anomalies	*IL11RA* NM_001142784.2	c.281 G > T p.(Cys94Phe)	Recessive	F	P	Late pancynostosis (4 y), papilloedema, hydrocephalus, midface hypoplasia, normal teeth
	Saethre–Chotzen	*TWIST1* NM_000474.3	c.309 C > G p.(Tyr103*)	Dominant, maternal	M	P	Crouzon-like appearance

*BC* coronal, *LC* left coronal, *LL* left lambdoid, *P* pancynostosis, *RC* right coronal, *S* sagittal.

^a^Analysed at an external laboratory, gene included in the custom-designed HTS panel.

^b^Affected suture: BC, LC, LL, P, RC, S.

We performed the analysis using the extended trio-based HTS panel on 22 individuals and revealed a diagnosis in 10; these were partitioned into 9 genetic syndromes, none of them commonly reported to include CS (Table 4). We detected two individuals with variants in the *AHDC1* gene, consistent with Xia–Gibbs syndrome. We further confirmed the following diagnoses: **c**oloboma, congenital **h**eart defects, choanal **a**tresia, **r**etardation of growth, developmental delay,** g**enital abnormalities, **e**ar abnormalities and deafness (CHARGE) syndrome, Bainbridge–Ropers syndrome (BRPS), CHDFIDD (Congenital heart defects, dysmorphic facial features, and intellectual developmental disorder, previously published [21]), Kleefstra syndrome, Genitopatellar syndrome, Floating–Harbor syndrome, Alpha-Mannosidosis (previously published [22]), and Malan syndrome (Table 4).

**Table 4:** Genetically confirmed diagnoses by the extended trio-based HTS panel.

Syndrome	Gene	Variant	Inheritance	Male/female	Suture^c^	Clinical features in line with the phenotypic description	Extension of phenotype
Xia–Gibbs syndrome	*AHDC1* NM_001029882.2	c.3185_3186del p.(Thr1062Serfs*63)	De novo	F	LC, S	Moderate developmental delay, autism, hypotonia, reduced vision, sleep disturbances	
Xia–Gibbs syndrome	*AHDC1* NM_001029882.3	c.2772del p.(Arg925Glufs*7)	De novo	M	M	Moderate developmental delay, short corpus callosum, hypotonia, short stature, proptosis, midface hypoplasia, long philtrum	Tethered cord, Chiari I malformation, omphalocele
Bainbridge– Ropers syndrome	*ASXL3* NM_030632.1	c.3033dup p.(Leu1012Serfs*23)	De novo	M	M	Moderate developmental delay, autism, reduced vision, feeding difficulties, sleep disturbances, strabismus, telecanthus, long philtrum, full lips, broad and proximally placed thumbs, behaviour difficulties	Craniosynostosis
CHDFIDD	*CDK13*^a^^,^^b^ NM_003718.4	c.2524 A > G p.(Asn842Asp)	De novo	F	M	Moderate developmental delay, autism, reduced vision, strabismus, proptosis, microcephaly, midface hypoplasia, broad nasal bridge, behaviour difficulties	Craniosynostosis
CHARGE syndrome	*CHD7* NM_017780.3	c.7593dup p.(Thr2532Aspfs*9)	De novo	M	S	Developmental delay, pulmonary atresia, VSD, cleft lip/palate, sensorineural hearing deficit, sleep apnoea, behaviour difficulties, feeding difficulties, scoliosis, micrognathia, hypotelorsim, cup-shaped ears	Late occurrence of craniosynostosis (5 years)
Kleefstra syndrome	*EHMT1* NG_011776.1 (NM_024757.4)	c.2018 + 1 G > C splice	De novo	M	S	Severe developmental delay, microcephaly, missing teeth, and delayed eruption, coarse facies, brachydactyly	Craniosynostosis
Genitopatellar syndrome	*KAT6B* NM_012330.3	c.3769_3772del p.(Lys1258Glyfs*13)	De novo	F	S	Knee flexion deformities, dislocated patella bilaterally, agenesis of corpus callosum, apnoea, hydronephrosis, severe eating difficulties, coarce facies, micrognathia, broad nose, died at 7.5 months of age	Craniosynostosis
Alpha-mannosidosis	*MAN2B1*^a^^,^^b^ NM_000528.3	c.1055 T > C p.(Leu352Pro)	Recessive	M	P	Intellectual disability, sensorineural hearing deficit	
Floating–Harbor syndrome	*SRCAP*^a^ NM_006662.2	c.7303 C > T p.(Arg2435*)	De novo	M	S	Developmental delay, short stature, hypertension, midface hypoplasia, deep-set eyes	Craniosynostosis
Malan syndrome	*NFIX* NM_002501.3	c.143 T > A p.(Met48Lys)	De novo	F	S	Moderate intellectual disability, macrocephaly, reduced vision, strabismus, long narrow face, deep-set eyes	Puberta praecox, craniosynostosis

*LC* left coronal, *M* metopic, *P* pansynostosis, *S* sagittal.

^a^Analysis performed at an external laboratory, gene included in the extended trio-based HTS panel.

^b^Previously reported.

^c^Affected suture: LC, M, P, S.

## Discussion

In our 18-year population-based cohort of children with CS, 27% fulfilled the presented clinical criteria and were diagnosed with syndromic CS. This is the highest number of syndromic cases reported from a population-based cohort and we believe the high genetic diagnostic yield of 84% supports the clinical criteria. We found a high level of genetic heterogeneity, with variants in common and well-known genes associated with CS accounting for 67% of the solved cases; the remaining cases were distributed across a diverse range of genetic syndromes, many of which are not commonly associated with CS.

We detected mosaicism in four families: one index individual and three healthy parents (Tables 2 and 3). A variant in the *EFNB1* gene was detected in a male with classic features of craniofrontonasal syndrome by HTS analysis. The variant presented as heterozygous in the analysis, suggesting mosaicism. The *EFNB1* gene is located on the X chromosome and loss-of-function variants in the *EFNB1* gene are assumed to cause craniofrontonasal syndrome through a paradoxical gender reversal in severity, where females usually develop typical features of craniofrontonasal syndrome and males usually have hypertelorism as the only feature. Random X-inactivation is assumed to be the cause of the severe phenotype in females, causing cellular interference as the cells have different expressions of EPHRIN-B1, generating abnormal tissue boundaries [23]. It has previously been proposed that males, being mosaic for variants in the *EFNB1* gene, will present with a severe phenotype, similar to females, due to the different expression of EPHRIN-B1, which is not tolerated [23]. Our results support this. We further detected low-grade mosaicism for a variant in the *FGFR2* gene in a healthy parent of two children with Crouzon syndrome and for a deletion (including the *TWIST1* gene) in a healthy father of a child with Saethre–Chotzen syndrome. Parental mosaicism for *FGFR2* and *TWIST1* variants is previously described [24, 25]. Crouzon and Saethre–Chotzen syndrome are inherited in an autosomal dominant manner, and this finding is important for genetic guidance, as it will impact the recurrence risk. Parental mosaicism for a *ZIC1* variant led to the variant initially being missed in the Trio-HTS analysis (filtering for de novo variants) in a boy with CRS6 and was only detected after manual re-evaluation of the gene due to his classical phenotype (Table 3). These cases demonstrate the need for a thorough evaluation of a well-described clinical phenotype, as diagnosis may be missed on trio analysis due to parental mosaicism.

We diagnosed syndromes not commonly associated with CS in 17 individuals, of whom 10 were detected by the extended trio-based panel and 7 by aCGH. We demonstrate an expansion of the clinical phenotype beyond CS in some cases (Table 4). Interestingly, all cases of rare syndromes detected by the extended trio-based panel, with two exceptions, had synostosis of a single midline suture only (Table 4). Likewise, seven out of ten microdeletion syndromes (Table 2) had midline synostosis only. This contrasts with the pattern typically seen in individuals with syndromic CS, where multiple suture synostosis is the most common finding [5, 26], and also with our finding in individuals with the more common CS syndromes (Tables 1 and 3). The most common reported CS syndromes have a high frequency of CS and are caused by genes acting in signalling pathways important for the development of the cranial sutures, mostly associated with osteogenic differentiation of stem cells (FGF/FGFR, Eph/Ephrin, TGFbeta/BMP, WNT) [27, 28]. The difference in affected sutures between the common CS syndromes and the rare or ultra-rare syndromes, with a low frequency of CS caused by genes acting in other pathways, might indicate that the synostoses in these two groups have different molecular mechanisms. Individuals with rare genetic syndromes which includes macrocephaly (e.g., Malan syndrome) might also be at higher risk of developing CS due to foetal head constraints that are associated with CS, especially regarding coronal premature fusion [27, 29].

Notably, in our cohort we detected several Mendelian disorders of chromatin modification (chromatinopathies), including (with the associated gene in parentheses): CHARGE (*CHD7*), Kleefstra (*EHMT1*), Floating–Harbor syndrome (*SRCAP*), KAT6B-related disorders (*KAT6B*), and 2q37 deletion syndrome (caused by haploinsufficiency of the *HDAC4* gene [30]). These genes influence the epigenetic machinery by targeting the DNA or the DNA-associated histone proteins, and variants that affect function are expected to have widespread epigenetic consequences [31, 32]. Approximately 44 chromatinopathies have been described to date. The most common mechanism is presumed to be haploinsufficiency, as a majority of the individuals have a loss-of-function variant [32]; this concords with our results (Tables 2 and 4). A few of the chromatinopathies have previously been associated with CS: Kabuki syndrome, Bohring–Opitz syndrome (BOS), and two cases of KAT6B-related disorders [31–36]. To our knowledge, only one case of CS in CHARGE syndrome [37], one case in Floating–Harbor syndrome [38], one case in 2q37 deletion syndrome [30], and none in Kleefstra syndrome have been reported. This study confirms CS as a feature of CHARGE syndrome, Floating–Harbor syndrome, KAT6B-related disorders, and suggests CS as a feature in Kleefstra syndrome and 2q37 deletion syndrome. We cannot be certain that haploinsufficiency of the *HDAC4* gene is the cause of CS in this case, as the individual also had a duplication on 11p15 in concordance with Silver–Russell syndrome. However, Silver–Russell syndrome is not associated with CS but rather delayed fontanelle closure. The presence of CS in several chromatinopathies at a low frequency adds to reports of other low-frequent malformations in these disorders. Their presence may be dependent on the molecular characteristics of the targeted genes, in addition to a general disruption of the epigenetic machinery; these are both suggested mechanisms for this phenotypic variability [31, 32, 39, 40]. Clinically, these findings suggest that individuals with chromatinopathies should be monitored for CS, in addition to other organ anomalies.

BRPS has phenotypic overlap with BOS. The former is caused by loss-of-function variants in the *ASXL3* gene and the latter by variants in the *ASXL1* gene. However, metopic synostosis, often seen in BOS, is not commonly reported in BRPS [41, 42]. Our case confirms that metopic synostosis is a rare feature in BRPS. CS has been reported in a very few individuals with CHDFIDD, Xia–Gibbs, Alpha-mannosidosis, and Malan syndrome [10, 43–45]. Individuals with Diamond–Blackfan anaemia have not been reported with CS.

Syndromic CS may be subdivided into syndromes with high risk of developing CS and a multitude of diverse syndromes usually defined by extracranial features with a low risk of developing CS. Due to the rarity of many syndromes, it is to be expected that the list defining the latter group is incomplete. Our results may point to a greater risk in subgroups of syndromes, such as the chromatinopathies.

Supported by our high diagnostic yield, we argue for the use of the presented clinical criteria, to ensure that all individuals with syndromic CS are identified, and thereby offered a broad genetic approach and assessment in a multidisciplinary team. For research purposes, a common clinical definition of syndromic CS is important to make reliable comparisons across cohorts. For some individuals, the features, indicating syndromic CS will not be present when the CS is evident. This argues for clinical follow-up after surgery for all individuals with CS. We recommend assessment of all individuals with syndromic CS in a multidisciplinary team to identify additional anomalies and progressive disturbances in facial growth, which may require repeat craniofacial surgeries [6, 7]. A high number of the syndromic cases in our cohort had a rare or ultra-rare genetic cause, mostly due to variants in different genes, emphasizing that syndromic CS is highly heterogeneous. This argues for a broad genetic approach. We suggest stepwise testing initiated by a custom-based HTS panel and aCGH, as the majority of the confirmed diagnoses were detected by these two analyses. In addition, our study showed that a number of variants were inherited from parents (including mosaics), all likely to be missed on the extended trio-based HTS panel. We then recommend trio-analyses, applying an extended panel of genes associated with development delay/anomalies in general, for negative cases. If the clinical presentation is highly suspicious of one of the frequent CS syndromes, one might consider testing the *FGFR2, FGFR3, TWIST1*, or *EFNB1* genes first; however, as this and other studies [46] have shown, a number of individuals have atypical presentations.

The main strength of the study is that the data are population-based and prospectively collected. Norway has an equal-access healthcare system that ensures a high inclusion rate. The unit is organized as a centralized multidisciplinary team, including a clinical geneticist. The clinical geneticist reassesses individuals initially diagnosed with nonsyndromic CS when new findings or difficulties present. A limitation of the study is that individuals diagnosed with CS over the last two or three years may not yet have presented with additional findings; thus, some syndromic cases may have been missed and the true number might be slightly higher. In syndromes not previously associated with CS, we cannot exclude the possibility of an additional genetic diagnosis associated with CS not detected by today’s methods (e.g., deep intronic variants). Newly associated genes, such as *SMAD6*, recently documented to be an important cause of CS [47], were not included in the panels. In addition, MLPA of *EFNB1* and *TCF12* were not available at our laboratory. According to this some diagnoses may have been missed. In addition, a few individuals included in the calculations were not analysed with the extended trio-based HTS panel (*n* = 3). This could mean that the genetic detection rate should be even higher.

## Conclusion

Using the presented clinical criteria, we identified one of the highest numbers of syndromic CS cases reported, strongly supported by a high genetic detection rate of 84%. The observed genetic heterogeneity and atypical presentations encourage a broad genetic approach in diagnosing syndromic CS. Surveillance for CS is recommended in a variety of genetic syndromes, including syndromes rarely associated with CS, such as the chromatinopathies, for the purpose of early diagnosis and treatment.

## Supplementary information

Custom-designed panel

Verified genetic diagnosis, gene variants and chromosome aberrations
